# Estimated Incubation Period and Serial Interval for Human-to-Human Influenza A(H7N9) Virus Transmission

**DOI:** 10.3201/eid2510.190117

**Published:** 2019-10

**Authors:** Lei Zhou, Qun Li, Timothy M. Uyeki

**Affiliations:** Peking University Health Science Center, Beijing, China (L. Zhou);; Chinese Center for Disease Control and Prevention, Beijing (L. Zhou, Q. Li);; Centers for Disease Control and Prevention, Atlanta, Georgia, USA (T.M. Uyeki)

**Keywords:** H7N9, incubation period, serial interval, human-to-human transmission, influenza, viruses, respiratory infections, China, zoonoses

## Abstract

We estimated the incubation period and serial interval for human-to-human–transmitted avian influenza A(H7N9) virus infection using case-patient clusters from epidemics in China during 2013–2017. The median incubation period was 4 days and serial interval 9 days. China’s 10-day monitoring period for close contacts of case-patients should detect most secondary infections.

As of April 2019, a total of 1,568 confirmed cases of avian influenza A(H7N9) virus infection acquired in China have been reported in humans since the virus emerged in spring 2013 (*1*,*2*). A large increase in infections occurred in China during the fifth H7N9 virus epidemic (2016–17), prompting concerns of increased H7N9 virus transmissibility in humans (*3*). However, as of June 2019, only evidence of limited, nonsustained human-to-human transmission has been reported (*3*).

Field investigations of case-patients with confirmed H7N9 virus infections are critical to assessing possible human-to-human transmission. The incubation period for H7N9 virus infection has been estimated to be 3–7 days (*4*–*6*). However, the incubation period estimated in these studies primarily reflects sporadic poultry-to-human transmission; no study has specifically focused on the incubation period for human-to-human H7N9 virus transmission. Although the kinds of exposures, amount of virus per exposure, and routes of exposure might differ between poultry-to-human and limited human-to-human H7N9 virus transmission, whether the incubation periods differ is unknown. Data on the incubation period for H7N9 virus in the setting of human-to-human transmission can help determine the appropriate duration for monitoring exposed close contacts, including healthcare personnel, of confirmed H7N9 case-patients.

We analyzed the data on all clusters of epidemiologically linked H7N9 case-patients collected during field investigations of 5 epidemics that occurred in mainland China during 2013–2017 and that were reported to the Chinese Center for Disease Control and Prevention (China CDC). We focused on clusters involving probable human-to-human transmission in which no poultry exposure, including visits to live poultry markets, was reported for epidemiologically linked secondary case-patients exposed to a symptomatic index case-patient, as previously described (*3*). We defined the incubation period for a secondary case-patient as days from the date of an unprotected exposure within 1 meter to an index case-patient for any duration beginning, at the earliest, the day before illness onset of the index case-patient to the date of illness onset of the secondary case-patient. The exposures and dates of illness onset were determined through field investigations of each H7N9 case-patient. For a secondary case-patient with multiple days of exposure to an ill index case-patient, we used the earliest exposure date to define the maximum incubation period and the last exposure date (such as the index case-patient’s date of hospital isolation) to define the minimum incubation period. We estimated incubation periods using median values, as done previously (*1*,*7*), and compared them by epidemic. We calculated serial intervals using the reported illness onset dates of index and secondary case-patients. We classified secondary case-patients as blood related or unrelated and compared median serial intervals by subgroup and epidemic.

Among 14 secondary H7N9 case-patients in 14 clusters of probable human-to-human transmission, the overall median estimated incubation period was 4 (range 1–12) days (Table). The median overall and medians of the minimum and maximum incubation periods estimated for secondary case-patients in the fifth epidemic were not significantly different than those estimated for case-patients in previous epidemics (Table). The estimated median serial interval among secondary case-patients who were blood related to an index case-patient (n = 6; 9.5 [range 5–12] days) and unrelated to an index case-patient (n = 8; 8 [range 6–15] days) were not significantly different (Appendix Table). The median serial interval for H7N9 virus infection among all clusters from the 5 epidemics was 9 (range 6–11) days and was not significantly different between epidemics (data not shown).

**Table:** Estimated incubation periods for avian influenza A(H7N9) virus infection in the setting of probable human-to-human transmission among 14 epidemiologically linked clusters of case-patients from 5 epidemic waves in mainland China, 2013–2017*

Incubation period, d, median (range); p value	Epidemic wave, no. secondary case-patients

All, n =14	First, n = 2	Second, n = 3	Third, n = 2	Fourth, n = 3	Fifth, n = 4
Overall	4 (1–12)	6.5 (1–12); 0.297	3.5 (1–7); 0.295	4.5 (1–7); 0.857	6 (1–11); 0.517	3.5 (1–8); 0.735
Minimum	1 (1–7)	3 (1–5); 0.830	1 (1–3); 0.519	2 (1–3); 0.914	1 (1–7); 1.000	2 (1–6); 0.581
Maximum	6.5 (3–12)	10 (8–12); 0.072	4 (4–7); 0.199	6.5 (6–7); 0.920	8 (5–11); 0.304	4 (3–8); 0.231

*After the first epidemic wave of infections, defined as March–August 2013, an epidemic wave was defined as September 1–August 31 of the following year. Thirteen secondary case-patients had multiple exposure dates and 1 secondary case-patient had 1 exposure date to an index case-patient. The incubation period for secondary case-patients was defined as the time in days from the date of an unprotected exposure within 1 meter to an index case-patient for any duration beginning at the earliest before illness onset of the index case-patient to the date of illness onset of the secondary case-patient. For secondary case-patients with multiple days of exposure to an ill index case-patient, we used the earliest exposure date to define the maximum incubation period and last exposure date (such as the date of hospital isolation of the index case-patient) to define the minimum incubation period. We compared the median incubation period for each epidemic wave with the 4 other epidemic waves. We used Wilcoxon rank-sum test to compare the distribution of median incubation periods; a p value <0.05 was considered statistically significant.

Overall, the incubation period and serial interval for limited human-to-human H7N9 virus transmission (including blood-related and unrelated persons) were unchanged during 2013–2017. Limitations to this study that could have affected our estimates include a small sample size of 14 secondary case-patients and the potential to misclassify secondary case-patients as a result of unrecognized or unreported poultry exposure. However, data on human, poultry, and environmental exposures and dates of illness onset included in our analyses were collected during detailed field investigations that were initiated promptly among close contacts of index case-patients after laboratory confirmation of H7N9 virus infection and reported to the China CDC.

Our use of median values to describe the epidemiologic parameters for H7N9 case-patients in the 14 clusters might have led to an overestimation of the incubation period and serial interval for human-to-human H7N9 virus transmission. For example, parametric analyses performed with data from much larger datasets (mostly H7N9 cases resulting from poultry exposures), in which data with right-skewed distributions were censored, were reported to provide shorter estimated incubation periods (*4*–*6*,*8*). The incubation period could also have been overestimated among case-patients with multiple exposure days to an index case-patient, if infection did not occur on the first day of exposure. Therefore, further comprehensive epidemiologic investigations to better define the transmission dynamics of human-to-human H7N9 virus transmission are critical. Nevertheless, our findings suggest that China’s policy since 2013 for a 10-day monitoring period for close contacts of H7N9 case-patients should detect most symptomatic secondary infections.

Appendix Estimated serial intervals for influenza A(H7N9) virus infection in setting of probable human-to-human transmission among 14 epidemiologically linked clusters of case-patients in mainland China, 2013–2017.
