# Supplementary material for: Estimated Incubation Period and Serial Interval for Human-to-Human Influenza A(H7N9) Virus Transmission
Source: Emerg Infect Dis. 2019 Oct;25(10):1982–3. doi: 10.3201/eid2510.190117 (PMC6759274; doi:10.3201/eid2510.190117)
Supplement: Appendix — Estimated serial intervals for influenza A(H7N9) virus infection in setting of probable human-to-human transmission among 14 epidemiologically linked clusters of case-patients in mainland China, 2013–2017. [file 19-0117-Techapp-s1.pdf]

Article DOI: https://doi.org/10.3201/eid2510.190117

Estimated Incubation Period and Serial Interval for Human-to-Human Influenza A(H7N9) Virus Transmission

Appendix

Appendix Table. Estimated serial intervals for influenza A(H7N9) virus infection in setting of probable human-to-human transmission among 14 epidemiologically linked clusters of case-patients in mainland China, 2013–2017*

Epidemic wave	Serial interval for clusters of blood-related contact, d, n = 6, median (range)	Serial interval for clusters of unrelated contacts, d, n = 8, median (range)	p value†
First	10 (8–12)		
Second	9	7.5 (6–9)	0.480
Third	10	7	ND‡
Fourth	5	12.5 (10–15)	0.221
Fifth	11	7 (6–11)	0.346
Overall	9.5 (5–12)	8 (6–15)	0.650

*The serial interval was defined as the time in days from illness onset of an index case-patient to the illness onset of a secondary case-patient in each cluster. A cluster of probable human-to-human influenza A(H7N9) virus transmission was defined as 2 or more epidemiologically linked cases in which a secondary case-patient had exposure to an ill index case-patient but no reported poultry exposure. ND, not done.

†Wilcoxon rank-sum test was used to compare the distribution of serial intervals, and $p<0.05$ was considered significant.

‡Only one blood-related cluster and one unrelated cluster occurred during the third epidemic wave.
